# Orthostatic Hyperkinetic Disorders in Subacute Sclerosing Panencephalitis – A Novel Observation

**DOI:** 10.5334/tohm.1277

**Published:** 2026-09-18

**Authors:** Sakoon Saggu, Deepa Dash, Animesh Das, Arunmozhimaran Elavarasi

**Affiliations:** 1 Department of Neurology, All India Institute of Medical Sciences, New Delhi, India; 2 Department of Clinical Neurological Sciences, University of Western Ontario, London, Canada

**Keywords:** Subacute sclerosing panencephalitis, SSPE, Orthostatic myoclonus, Orthostatic hyperkinetic disorders, Orthostatic Tremor

## Abstract

**Background:**

Subacute sclerosing panencephalitis (SSPE) is a progressive, often fatal inflammatory encephalitis caused by persistent measles virus infection of the central nervous system. The neurological manifestations of SSPE are well characterised, encompassing cognitive decline, periodic myoclonic jerks, and often relentless progression.

**Case Report:**

We report a case of a 14-year-old male with SSPE who developed bilateral lower limb shaking and knee buckling, with associated loss of quadriceps tone on standing. These features resulted in recurrent falls and necessitated supported ambulation.

**Discussion:**

To our knowledge, this combination of orthostatic limb shaking and loss of tone in the context of SSPE has not been previously described in the literature. This case expands the recognised phenotypic spectrum of SSPE.

## Introduction

SSPE is a rare and often fatal neuro-inflammatory condition affecting predominantly children and adolescents, arising from persistent defective measles virus infection years after the initial exposure. The clinical course [1,2] follows a well-recognised progression: initial behavioural and intellectual deterioration, followed by myoclonic jerks, seizures, and eventual autonomic dysfunction and a vegetative state. While myoclonus is a hallmark of SSPE, the occurrence of orthostatic hyperkinetic movement phenomenology, namely orthostatic lower limb shaking and orthostatic negative myoclonus, represents an atypical and underreported manifestation. We present a case illustrating these phenomena and their functional consequences.

## Case Presentation

A 14-year-old unvaccinated male, had developed measeles at the age of 7 years, with a self limiting course over two weeks and presented with a one-year history of falls and increasing difficulty with standing. His caregivers reported that he had experienced cognitive decline relative to his premorbid baseline, after a six year latent period following the initial measles infection; however, he retained the ability to obey simple commands and remained functionally independent in activities of daily living. He was ambulatory with physical support.

**Figure 1:**
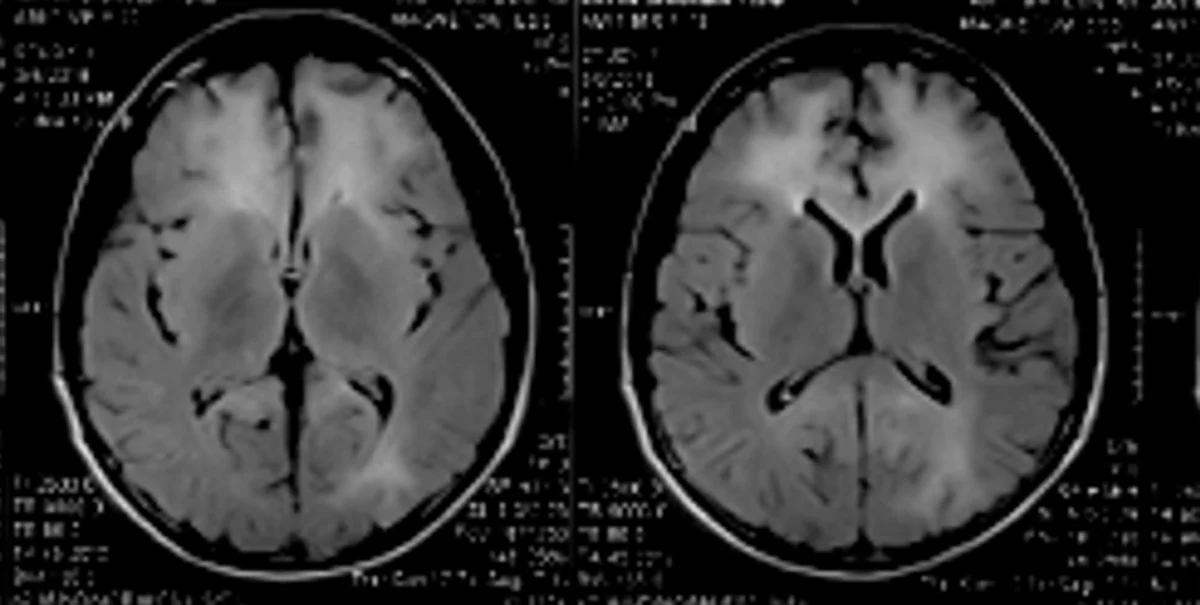
Brain MRI showing bilateral frontal grey and white matter lesions with involvement of the genu of the corpus callosum.

On clinical assessment, the patient demonstrated shaking of the lower extremities that appeared exclusively on standing (Video 1) and resolved upon sitting, lying down, and walking. In addition, brief, sudden episodes of bilateral knee buckling were observed during the orthostatic position and persisted during walking, manifesting as transient knee flexion interrupting the gait cycle, characterised by loss of quadriceps tone without preceding tonic activity, consistent with negative myoclonus. These episodes were the principal cause of his recurrent falls. The orthostatic phenomena were reproducible on examination. Imaging showed bilateral frontal grey and white matter lesions with involvement of the genu of the corpus callosum (Figure 1). There were no brainstem lesions.

**Video 1:** The video demonstrates orthostatic lower limb shaking and orthostatic negative myoclonus in a 14-year-old male with SSPE. On standing, shaking is evident in both lower limbs, transmitted to the trunk and upper body. Superimposed on this, episodic bilateral knee buckling with giving way is observed, consistent with orthostatic negative myoclonus. On walking, the shaking resolves; however, periodic episodes of negative myoclonus persist, manifesting as brief interruptions of gait with transient knee flexion.

He had Jabbour Stage II symptoms and was confirmed to have SSPE based on an EEG showing periodic complexes, MRI with subcortical white matter involvement, and CSF measles index positivity. He was treated with intrathecal interferons and valproate. There was stabilization of his symptoms over a follow up of 6 months.

## Discussion

Orthostatic tremor is a distinct movement disorder characterised by a high-frequency (13 to 18 Hz) tremor of the legs and trunk upon standing, with symptomatic improvement on walking or sitting [3]. It has been associated with different etiologies, including Parkinson’s disease, dystonia, and other movement disorders [4,5,6,7]. Orthostatic negative myoclonus, by contrast, involves brief lapses of postural muscle tone, clinically manifesting as sudden knee buckling or “leg giving way” on standing. Both phenomena have been reported in various neurological contexts, including cortical and subcortical disease [8]. There are no reports of their co-occurrence in SSPE.

There is evidence to support staging-dependent shift [9] in SSPE myoclonus subtype, with cortico-subcortical involvement in Jabbour II before progressing to brainstem-predominant myoclonus at later stages. This cortico-subcortical generator is consistent with a network capable of producing both standing-triggered oscillatory activity and inhibitory cortical lapses, selectively unmasked by postural loading. The cerebellar–SMA pathway implicated in primary OT may be rendered dysfunctional by SSPE’s predilection for subcortical white matter, producing conditions analogous to the double-lesion hypothesis in which concurrent cerebellar and basal ganglia disruption permits the orthostatic oscillator to become clinically manifest, a mechanism supported by the precedent of orthostatic leg tremor arising from mGluR5 autoimmune encephalitis [10].

One case report documents orthostatic leg tremor as the initial manifestation in mGluR5 autoimmune encephalitis, suggesting that inflammatory CNS disease can generate orthostatic-specific motor phenomena [10].

In this patient, the combination of orthostatic limb shaking and orthostatic negative myoclonus likely reflects disruption of the subcortical and cortical networks subserving postural tone regulation, consistent with the diffuse encephalitic process of SSPE. The selective involvement of the quadriceps during weight-bearing activities (both standing and walking), with preservation of sufficient motor function for supported ambulation and independence in ADL, suggests a functional threshold effect in which postural loading unmasks deficits that are not apparent at rest.

The principal limitation of this case is the absence of surface or needle EMG, which is the gold standard for confirming a diagnosis of orthostatic tremor and characterizing the frequency and identifying the electromyographic silent periods pathognomonic of negative myoclonus. Thereby, we were not able to categorize the limb shaking as tremor because of the absence of electrophysiologic studies. Future cases should incorporate neurophysiological assessment to more precisely characterise these phenomena.

## Conclusion

This case expands the recognised phenotypic spectrum of SSPE to include orthostatic limb shaking and orthostatic negative myoclonus. Clinicians evaluating young patients with falls, particularly in the context of cognitive decline or in measles-endemic regions, should consider SSPE in the differential. Electrophysiological evaluation with EMG is recommended when feasible to confirm the diagnosis.
